# Reducing Close Encounters with Insect Pests and Vectors: The Past, Present and Future of Insect Repellents

**DOI:** 10.3390/insects17020130

**Published:** 2026-01-23

**Authors:** Luis A. Martinez, Laurence J. Zwiebel

**Affiliations:** Department of Biological Sciences, Vanderbilt University, Nashville, TN 37235, USA; luis.a.martinez.2@vanderbilt.edu

**Keywords:** repellents, disease vectors, agriculture, insecticides, olfaction, VUAA1

## Abstract

Insects acting as agricultural pests or disease vectors represent some of the greatest challenges to global health, food security and economics. Diverse technologies to combat insects of economic and medical importance have been and are continually being developed. These include natural and synthetic chemical insecticides and repellents, mass-trapping approaches and, more recently, an increasingly wide range of biological as well as genetic manipulations of insect vectors/pests. The increase in biological resistance and cross-resistance to many insecticides and repellents, the rapid expansion of human populations, as well as escalating climate change have extended or shifted the active periods and habitats of many insect species, creating new hurdles for attempts to defend humans from insects. At the same time, environmental, ecological and socio-political concerns continue to impact the utility of both current interventions as well as newly emerging innovative strategies. The near exponential increase in insect-based threats highlights the importance of basic and translational studies to design and develop novel technologies to combat detrimental insect populations. This review outlines the history of these challenges and describes the evolution of chemical insect control technologies, while highlighting existing and contemporary approaches to develop and deploy chemical repellents to address this threat to human health and agriculture.

## Simple Summary

Reducing close encounters with insects remains one of the most important efforts to protect humans from hunger and disease. Understanding the history and biology of chemical insect control strategies paves the way toward developing new long-lasting, safe and effective approaches.

## 1. Introduction

Many insects have evolved to rely on parasitic and micropredatory relationships with humans, especially after the shift toward establishing permanent human settlements, which arose during the Neolithic Revolution between 8000 and 10,000 BCE. During that time, each has used the other as both a food/nutrient source [1,2] and in the context of agriculturally specific interactions around food production and storage [3]. Since then, the interactions between humans and insects—with both beneficial insects such as pollinators and less beneficial and indeed harmful insects such as agricultural pests and disease vectors—have been extremely impactful at nearly every level of consideration. While anthropophilic and hematophagous arthropods have doubtlessly plagued humankind since the origin of our species, the systematic use of chemical strategies to control pests likely arose with the start of agricultural practices. The movement from nomadic lifestyles to agricultural settlements, which included food storage, farming and livestock, led to new and powerful associations between humans and what was until then agricultural pests, such that some insect species, e.g., the German cockroach (*Blattella germanica* L.) and the granary weevil (*Sitophilus granarius* L.), are now only found peri-domestically with humans [4,5,6].

It is reasonable to posit that the need to protect agricultural crops and livestock from the threat of harmful insects was the driving force for the earliest forms of insect control strategies that arose in the form of religious and superstitious ritual practices [7,8]. The most successful of these rituals, which unwittingly but undoubtedly must also have had a biological basis for efficacy and therefore relevance, became more commonly adopted and eventually led to the development of ancient insecticides and repellents, such as plant-derived extracts, ash, smoke and various mineral toxins (including sulfur and arsenic) [7,8,9].

Several iterations of increasingly more effective strategies—such as pyrethrum extracts from chrysanthemum plants, arsenical insecticides including Paris Green (copper acetoarsenite) and lead arsenite, and hydrogen cyanide fumigant gas—were developed over the next few millennia [10,11,12,13]. However, these advances came about slowly so that until the advent of modern chemistry roughly 100–150 years ago a wide range of insect pests regularly devasted crop yields [14,15]. The discovery in the late 1800s and early 1900s that malaria and other pathogens were transmitted by mosquitoes and other insects spurred efforts to identify and develop biological and chemical strategies to control insect vectors [16,17,18]. These efforts focused primarily on the development and widespread use of insecticides such that between the late 1930s and the 1960s the number of identified insecticides increased exponentially [19,20,21,22]. Subsequently, the devasting impact of disease vectors on troops during World War II generated great breakthroughs across several classes of chemical-based insect control actives that were later deployed on massive scales in agricultural contexts [23,24].

The first warnings of insecticide resistance were raised as early as 1914 with the San Jose scale (*Comstockaspis pernicosa*) tolerance against sulfur lime spray and lead arsenate resistance in gypsy moths (*Lymantria dispar*; ref. [25]). Since then, along with the long-term ecological and environmental effects of widespread insecticide use [22,26,27,28], there has been a steady rise in insecticide resistance and cross-resistance. Strategies to manage or delay the development of insecticide resistance have included scheduled rotations of chemicals and the use of large targeted initial doses. However, the widespread use of sub-lethal residual concentrations and cross-resistance to actives with shared modes of action (MOAs) have, ultimately, exponentially accelerated the global levels of insecticide resistance [21,26,29]. These collateral effects highlight the utility of non-lethal strategies, which inherently pose substantially lower risks of losing efficacy due to resistance. In that context, it can be argued that the rapid rise in resistance to the current repertoire of chemical-based insecticide strategies necessitates the discovery of novel long-lasting compounds with distinct molecular targets from existing chemicals.

## 2. History and Evolution of Chemical Strategies

### 2.1. Insecticides

Among the earliest means of pest control was the use of various minerals, such as the heavily reported use of sulfur compounds by ancient Sumerians ~2500 BCE to control insects and mites, as well as the use of mercury and arsenic in ancient China ~1200 BCE [7]. Sulfur was used by both ancient Greeks and Romans, and across Asia, as early as 1000–200 BCE, while the deployment of arsenic and other heavy metals in China has also been documented as early as 900 CE [8,30,31]. While they are often accompanied by dangerous off-target effects toward humans and other mammals, these chemical approaches persisted through antiquity and into the modern age because of their high efficacy [30]. Arsenic, antimony, selenium, sulfur, thallium, zinc and copper remained among the primary actives of most insecticides well into the 1930s [30,32]. Not surprisingly, the use of many of these mineral-based chemicals is currently highly restricted or banned outright by various agricultural, environmental and other regulatory agencies in both developed and developing nations [33,34].

**Figure 1:**
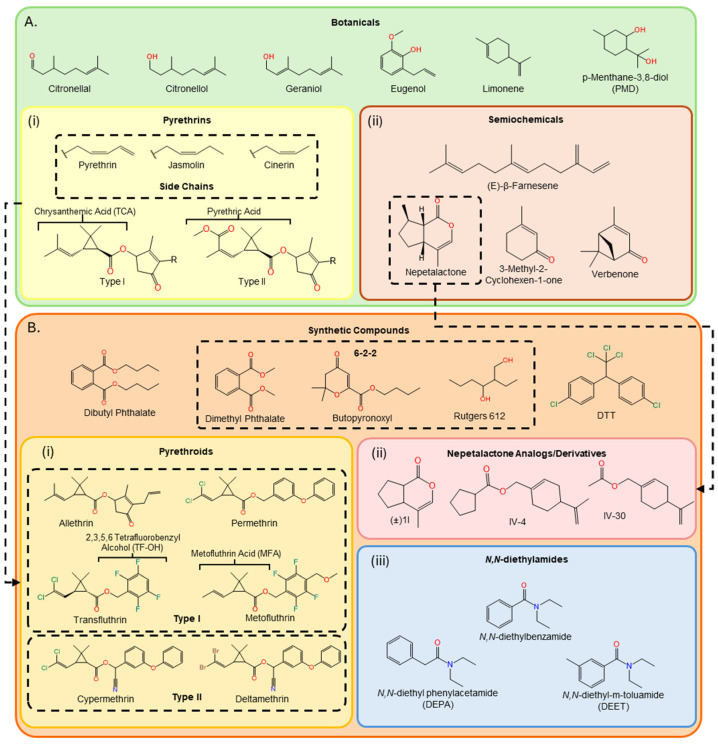
Chemical structures of botanical and synthetic insect control compounds. (**A**) Botanical agents are naturally occurring and plant-derived compounds, typically produced by host plants to deter pests and can be used in insect control strategies. Many botanical repellents are volatile terpenes and terpenoids with high volatility and low durability. Pyrethrins (i) are a particularly effective class of botanicals that affect the nervous system by targeting voltage-gated ion channels. Semiochemicals (ii) act as pheromones and allelochemicals to modulate insect behaviors and can function as attractants or repellents via sex, threat and anti-aggregation signals. (**B**) Synthetic agents can include unique compounds, such as DDT, as well as compounds derived from botanicals or other naturally occurring chemicals. Pyrethroids (i) are synthetic pyrethrin analogs that have significant repellent, insecticidal and stability advantages over natural pyrethrins. Nepetalactone analogs (ii) are synthetic semiochemicals derived from the aphid sex pheromone to repel aphids [40]. N,N-diethylamides (iii) are a unique class of actives developed for insect control, which includes DEET, the most commonly used ingredient in commercial repellents.

Plant-derived compounds, known collectively today as “botanicals” (Figure 1A), were also among the earliest forms of chemical-based insect control and were used as early as 1200 BCE in ancient China and 1550 BCE in ancient Egypt, according to the Ebers Papyrus which discussed the use of both chemical and biological substances [7,35]. Greek and Roman sources also reported the use of plant burning to fumigate crops and treat insect infestations [8]. Pyrethrum, an extract of *Chrysanthemum roseum* flowers and dried chrysanthemum flowers, was used to dust crops throughout the Middle Ages in China and Iran and eventually became popular in Europe [36]. The biochemically active components of pyrethrum were ultimately identified in 1923 as a mixture of chrysanthemic and pyrethric acid esters and classified as type-I and -II pyrethrins, respectively (Figure 1(Ai)). Both type-I and type-II pyrethrins occur in three natural variations: pyrethrins I and II, jasmolins I and II, and cinerins I and II (Figure 1(Ai)). While similar in structure, esters of chrysanthemic acid and pyrethric acid are differentiated by the presence of an additional methyl ester group on pyrethric acid. In practical terms, a major impediment for the agricultural use of pyrethrins is their low photostability and duration of activity. Modern pyrethroids are a major class of synthetic pyrethrin analogs that were developed to overcome these limitations and deliver even greater insecticidal potential. Similarly to natural pyrethrins, synthetic pyrethroids are categorized into type-I and -II molecules that are differentiated by the presence of a cyano group on the alpha-benzylic position of the type-II molecule (Figure 1(Bi)). Synthetic pyrethroids, such as permethrin and deltamethrin, address the limitations of natural pyrethrins by increasing both the stability of the molecules and their neurotoxicity to insects. Both natural pyrethrins and synthetic pyrethroids remain major classes of chemical insecticides in many contemporary insect control strategies. In fact, pyrethroids account for up to one third of global pesticide actives with an estimated market of 3.74 billion USD as of 2024. That market is predicted to rise to 3.91 billion USD in 2025 [37], highlighting the economic significance of these insecticides in global economies.

The organochloride dichloro-diphenyl-trichloroethane (DDT; Figure 1B), which opens neuronal sodium ion channels in both insects and mammals, was identified as a highly effective broad-spectrum insecticide by Paul Hermann Müller during WWII and quickly became the standard for insect control due to its high efficacy against most insects at low concentrations [23,38]. There is little dispute that DDT saved countless lives throughout WWII and into the 1950s, when over 2.5 million people were treated with it against lice to prevent the spread of typhus. In the late 1940s, the use of DTT was approved for agricultural purposes following WWII and it was implemented for defending crops against pest insects. However, the widespread and irresponsible use of DDT in food production and vector control, which peaked in 1961, caused long-standing ecological and environmental damage, negative human health implications and concerns of resistance, which resulted in its agricultural ban and fall in popularity in the 1970s [38,39].

### 2.2. Repellents

In addition to the near canonical use of insecticides, the early 20th century also gave rise to the discovery and development of several non-lethal but nevertheless highly efficacious synthetic insect repellents. Among the first of these were dialkyl phthalates (Figure 1B), which were identified as insect repellents by Warren Moore and Hyym E. Buc in 1928; butopyronoxyl (commercially known as Indalone^®^), which was discovered by Lowell B. Kilgore in 1936; and 2-ethyl-1,3-hexanediol (Rutgers 612) discovered by Philip Grannett and Harry L. Haynes at Rutgers University in 1942 [41,42,43,44,45]. Dialkyl phthalates, predominantly dimethyl (DMP) and dibutyl phthalate (DBP, Figure 1B), are organic solvents and plasticizers for cosmetic, pharmaceutical and industrial products. These actives demonstrated significant efficacy in early repellent products [46]. The loss of repellent activity observed in some formulations was attributed to the chemical incompatibility of dialkyl phthalates with other actives in formulation [47,48]. While individually repellent, three of these compounds were formulated during WWII into a single, highly effective, topical repellent known as 6-2-2, denoting its composition of six parts dimethyl phthalate, two parts Indalone and two parts Rutgers 612 (Figure 1B) [41]. While effective at deterring host-seeking arthropods from interacting with human hosts, these compounds required direct application on skin or clothing and were later discovered to have toxic health implications, such as endocrine disruption and teratogenicity [48].

In 1942, in the search for safer and more effective chemical actives to address harmful insect populations for use by the United States Army, the United States Department of Agriculture (USDA) screened more than 7000 compounds for insecticidal, miticidal and repellent effects [41,49]. This effort identified N,N-diethylbenzamide and, subsequently, several of its chemical derivatives, such as highly potent N,N-diethylamide-based repellent compounds N,N-diethyl phenylacetamide (DEPA) and N,N-diethyl-m-toluamide (DEET; Figure 1(Bii)) [41,50]. Currently, DEET remains the most commonly used active ingredient (AI) in commercially available insect repellent formulations, with several alternative hypotheses for its putative MOA(s) [51], as outlined below.

## 3. The Biological Bases of Chemical Repellents

**Figure 2:**
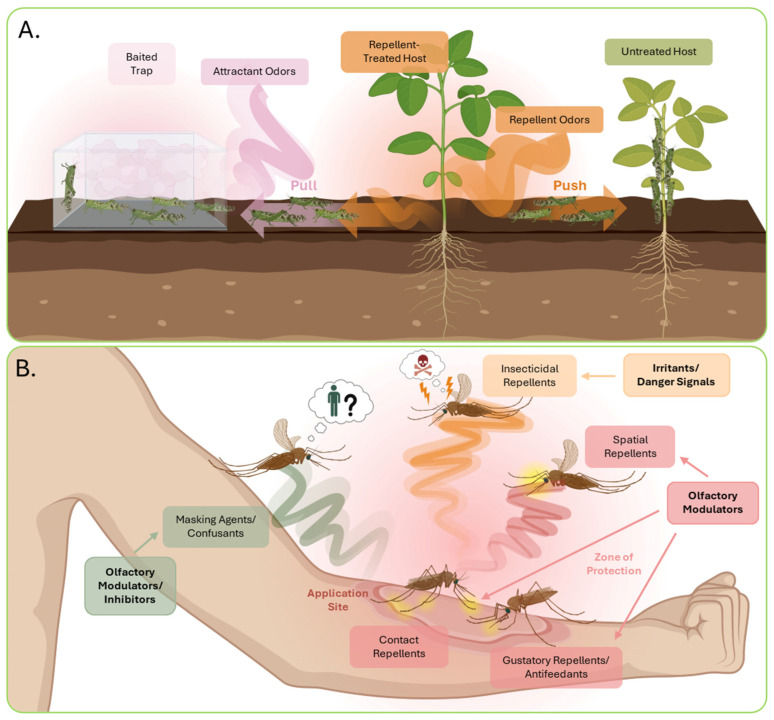
(**A**) Example of a push–pull strategy using volatile repellents to “push” grasshoppers away from a repellent-treated host plant in combination with an attractant-baited trap to “pull” and capture them. Untreated plants remain unprotected from pests. (**B**) Repellent compounds are categorized by the mechanisms by which they disrupt insect behavior. Contact repellents and antifeedants are non-volatile compounds that require direct contact at the application site. Contact repellents target the chemosensory appendages, such as the tarsi, while antifeedants specifically target the mouthparts during insect–host interactions to disrupt food-seeking behavior. Spatial repellents are volatile compounds that deter insects from interacting with host species from long distances providing a zone of protection around the host. Insecticidal repellents are acutely toxic compounds that repel insects by triggering defensive behaviors in response to harmful or painful stimuli. Masking agents and confusants interfere with chemosensation through receptor modulation or preventing volatilization of host odors.

While formally classified as insecticides by the United States Environmental Protection Agency (US-EPA), most chemical repellents represent non-lethal alternatives to traditional insecticides for the control of insects of economic and medical importance. Acting through a diverse spectrum of MOAs, repellents interfere with the ability of insects to locate and interact with animals and plants that they typically target as food sources and host organisms. Additionally, the impact of repellent chemistries can be amplified with the accompanied use of baited traps containing attractive odor formulations, typically known as “push–pull strategies” (Figure 2A) [52]. Push–pull strategies, in which plants treated with deterrent compounds “push” insects away from their desired hosts while a nearby baited trap “pulls” and captures the insects to prevent future encounters, have been effective to protect food crops from pest insects [53], such as the specific case of intercropped repellent plants used to repel stemborers from maize while nearby attractive plants are used as bait [54]. Repellents can be characterized by both the substrate through which they accomplish their deterrent activities and their mode of delivery. While the precise molecular targets and MOAs for many repellents remain topics of study and debate, contact and gustatory repellents are non-volatile and require direct physical interaction with insects while spatial repellents are typically volatiles that can interact with insects over long distances (Figure 2B). Interestingly, the term repellent has also been used to describe compounds that interfere with the detection of host odors, either by masking the host odors or the inhibition of chemosensory processing (Figure 2B).

### 3.1. Olfactory Modulators

The MOA controversy surrounding repellents is best illustrated by DEET, for which various mechanistic functions have been proposed. Many studies have concluded that DEET functions by targeting members of diverse insect chemosensory receptor families through a varied spectrum of hypothetical mechanisms [55,56]. These include (i) the direct activation of specific olfactory receptor neurons (ORNs) to generate aversive/deterrent behaviors independent of the presence of attractant odors [57,58]; (ii) the direct inhibition of ORNs that blocks canonical responses to attractive odorants and prevents host identification, such as attenuating the detection of L-lactic acid, carbon dioxide and 1-octen-3-ol [59,60,61]; (iii) the modulation of odorant receptor (OR) complexes that results in confused odor perception [62]; (iv) direct chemical interactions with host odors, reducing their volatility and thereby their interaction with insect sensory systems [63]; (v) the modulation of gustatory receptor (GR) neurons (GRNs), specifically the activation of S-type GRNs and the inhibition of L-type GRNs, to stimulate bitter taste detection and block sweet tastes [64,65]. Moreover, there is evidence suggesting that DEET targets different chemosensory receptor classes in a species-specific manner, as Aedine mosquitoes are targeted through OR/Orco-dependent pathways while Anopheline mosquitoes appear to be targeted through ionotropic receptor-(IR) and GR-dependent pathways [66]. These varying physical and physiological pathways illustrate the many putative mechanisms by which DEET may (or may not) function, as well as the underlying MOAs of most currently recognized repellent chemistries. Given the many proposed MOAs explaining the repellency of DEET observed in insects and other arthropods, it is very likely that this multi-modality is indeed the reason for its efficacy. While OR/Orco-dependent mechanisms have been shown to contribute to DEET repellency, it must also be noted that DEET effectively repels non-insect arthropods, necessitating the existence of other chemosensory pathways, such as through GRs or IRs.

### 3.2. Insecticidal Repellents

In contrast to non-lethal AIs, such as olfactory modulators, several acutely toxic insecticidal compounds also function as aversive insect repellents when administered directly at sublethal concentrations or as long-distance volatiles [67]. These actives function primarily as insecticides but are hypothesized to also act as excito-repellents by directly overstimulating the insect nervous system. Both DDT and pyrethrins/pyrethroids are neurotoxic insecticides that target voltage-gated sodium channels throughout insect nervous systems that share structural and functional homologies with the alpha subunit of mammalian sodium channels [39]. These AIs affect insect central nervous systems by preventing voltage-gated sodium channels from closing, thus maintaining a state of hyperexcitability that induces paralysis and knockdown after prolonged or high-concentration exposure. As a secondary mode of action, these AIs also target insect peripheral nervous systems by stimulating neurotransmitter release at neuromuscular junctions, which leads to neurotransmitter depletion, excitatory paralysis and death. While type-I and type-II pyrethroids are highly similar in structure and function, type-II pyrethroids are more effective at killing insects by inducing long-lasting irreversible depolarization in axons and nerve cell terminals. In contrast, type-I pyrethroids typically require larger doses, leading to higher AI concentrations, and/or more prolonged exposures to achieve lethality. While less toxic to mammals than insects, DDT, pyrethrins and pyrethroids similarly affect the mammalian central and peripheral nervous systems to induce tremors, choreoathetosis, salivation and clonic seizures [68,69]. The shared molecular target of DDT and pyrethroids, and their prevalent use since the 1950s, has led to widespread resistance and cross-resistance to both chemical classes and other chemistries that target sodium channels [70]. One novel development in pyrethroid-derived repellent chemistries demonstrated the efficacy of pyrethroid acids and alcohols (Figure 1B(i)) against resistant Aedine strains [71]. Moreover, these compounds elicited synergistic vapor toxicity effects on mosquitoes with pyrethroids, as well as distinct mammalian toxicity effects different from those of pyrethroids. These findings suggest that pyrethroid-derived acids and alcohols may have discrete molecular targets, different from those of the parent molecule. Importantly, this finding also indicates that novel and effective chemical repellents could be derived from other existing repellent compounds to mitigate the impact of resistance and increase efficacy.

### 3.3. Novel Chemical Repellent Classes

Concerns over the rise in resistance to our current repertoire of chemical insect control agents, along with the growing threat of climate change-driven insect population growth, have fueled research interest in developing new classes of non-lethal repellent AIs with unique MOAs. Among the lead compounds being investigated for use as control agents against agricultural pests and disease vectors are cyclic acetals, cellulose nanocrystals, graphene oxides, para-pheromones and Vanderbilt University Allosteric Agonists (VUAAs). These novel and unique chemistries aim to address the limitations, that is toxicity, repellence, stability and duration, in the current suite of available insect control agents.

Terpenoids are naturally occurring volatile botanicals that function in various defense mechanisms of plants. Structure–activity relationship (SAR) investigations of synthetic terpenoids for insect control strategies have aimed at generating analogs with improved repellent and insecticidal utility by targeting the inherent limitations of these AIs. A major challenge of terpenoids is their high volatility and low duration of action. One approach to reduce the volatility of monoterpenoids, including thymol and citronellol, has been to synthesize esterified monoterpenoid analogs of greater molecular weight. While studies did not find correlational effects between molecular weight and insecticidal efficacy, many analogs have demonstrated improved temporal efficacy [72,73]. Additionally, novel synthetic cyclic acetal and hydroxyacetal analogs of the terpenoids menthone and citronellal have improved repellency by decreasing their volatility and, thereby, increasing their stability and duration [74]. Terpenoid derivatives and entirely novel, synthetic cyclic hydroxyacetals were shown to have repellencies comparable with and/or better than DEET and icardin, while having longer effect durations and lower levels of epithelial permeation [74,75].

Graphene oxides, cellulose nanocrystals and other novel, two-dimensional particle/platelet formulations are being investigated to reduce the blood feeding of hematophagous insects by decreasing the volatilization of host odors while simultaneously creating barrier films on skin surfaces so that they cannot be pierced by insect mouthparts [76]. While this research is still in its infancy, current studies aim to overcome the limitations of graphene oxides and other wearable chemical barriers, such as stability under varying environmental conditions. Evidence from exploratory laboratory studies supports that such strategies can be used to reduce insect biting frequencies [77].

Interest in semiochemical-based strategies has also increased in recent years due to several advantages over chemical alternatives. Semiochemicals, categorized as either pheromones (intraspecies) or allelochemicals (interspecies), are volatile, naturally occurring, biologically relevant molecules, which have the potential for use in the development of repellents, as well as attractants, with specificity against only target insects along with lower risks of negative environmental and ecological impacts and resistance development. Para-pheromones are synthetic pheromone mimics and analogs that can provide greater efficacy and stability than naturally occurring analogs. Synthetic anti-aggregation and alarm pheromones, such as verbenone, 3-methyl-2-cyclohexen-1-one (MCH) and (E)-β-Farnesene (EBF), have been implemented as effective repellents against lumber and agricultural pests, such as bark beetles and aphids (Figure 1(Aii)) [78,79,80]. Recent studies have demonstrated the efficacy of novel para-pheromones for agricultural use in both laboratory and field settings. Synthetic analogs of nepetalactone (Figure 1(Biii)), a plant-derived aphid sex pheromone, were recently shown to have increased stability and stronger repellent effects than their naturally occurring counterpart in laboratory-based olfactometry assays [40]. In these assays, nepetalactone-derived chemistries were synthesized to mimic the effects of the alarm pheromone EBF to repel aphids from food sources. In contrast, sex signaling compounds, such as the allelochemical methyl eugenol, have been used in recent field studies as effective attractants in baited traps in combination with different insecticides to capture mango fruit flies [81].

**Figure 3:**
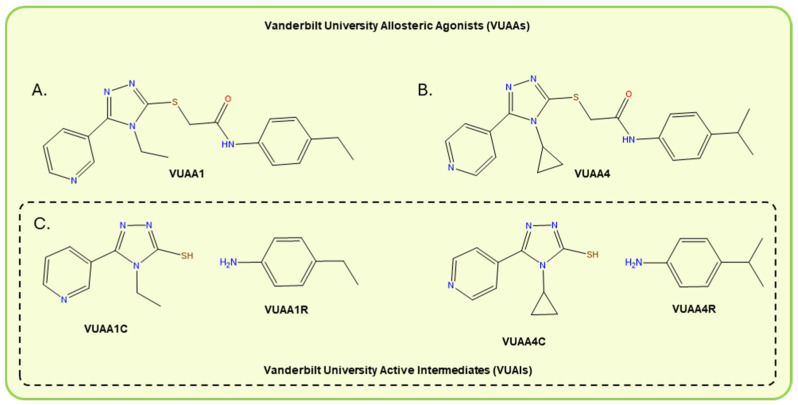
Chemical structures of Vanderbilt University Allosteric Agonists VUAA1 (**A**) and VUAA4 (**B**). (**C**) Chemical structures of Vanderbilt University Active Intermediates: VUAA1C, VUAA1R, VUAA4C and VUAA4R.

VUAAs are a novel class of synthetic molecules (Figure 3) that specifically agonize the highly conserved and obligatory insect odorant receptor coreceptor (Orco) and, consequently, are hypothesized to induce the widespread activation of insect ORNs [82,83]. Insect OR complexes comprise two primary subunits in tetrameric ligand-gated ion channel configurations [84,85]. The tuning OR subunits (ORX’s) are responsible for ligand-specific ORN activation and allow for the recognition of environmental chemical cues. Orco is required for membrane transport and ion channel formation, and recent in vitro subunit stoichiometry studies revealed that the Orco subunit makes up three-fourths of the functional OR-Orco heterotetrameric complex [84,85,86,87]. Moreover, Orco is found in nearly all insect species and has remained remarkably conserved among taxa, in many cases sharing over 90% amino acid identity within insect families and about 60% identity between orders of the insect class [88,89,90,91]. Thus, Orco-targeting actives such as VUAAs have the potential for use as broad-spectrum insect repellents in both agriculture and vector control via the aversive hyperstimulation of the insect olfactory signaling pathway, which is molecularly distinct from vertebrate olfaction [82,83,89]. VUAA1 (Figure 3A) was the first allosteric Orco agonist identified through the high-throughput screening of a small molecule library of 118,720 compounds using human embryonic kidney (HEK293) cell lines expressing exogenous *An. gambiae* (Ag) Orco in combination with various AgORXs [82]. VUAA1 elicited electrophysiological responses from all HEK293 cell lines regardless of which AgOrco + AgORXs they expressed as well as cell lines containing only AgOrco or *D. melanogaster* Orco [82,83,86]. SAR analysis of the VUAA1-Orco interaction revealed three novel VUAA structures with greater agonist activity than VUAA1, the most potent of which is VUAA4 (Figure 3B), as well as several antagonists, analogs with lesser agonist activity, and many completely inactive analogs [92,93,94]. The results of these SAR studies also demonstrated the dramatic impacts of very small structural changes to the VUAA1 molecule, highlighting the tight specificity of VUAA-Orco interactions [92,93].

While the targeting of Orco by VUAAs foreshadows its utility in broad-spectrum insect control strategies, the difficulty to volatilize these compounds has placed obstacles in its development in spatial repellent formulations [82,95,96]. The thermal volatilization of lead VUAAs increases their spatial efficacy through the release of volatile thermolytic degradation, products which we have named VUAA-based active intermediates (VUAIs; Figure 3C) [95]. Binary mixtures of VUAIs have demonstrated increased spatial repellency against Anopheline mosquitoes when compared with VUAAs and DEET in laboratory behavioral assays [95]. VUAIs have been postulated to be able to recapitulate the allosteric agonist activity of parent VUAA molecules by synergistically interacting with active sites in the Orco subunit. However, it is evident that, similar to pyrethroid-derived acids and alcohols, VUAIs may interact more promiscuously with chemosensory pathways than VUAAs and possibly have other modes of repellency [95]. This is exemplified by the repellency observed from VUAIs in Orco loss-of-function mutants, which may be explained by interactions with other olfactory receptors families (GRs/IRs) or acute toxicity.

## 4. Contemporary Repellent Formulations in Agriculture and Vector Control

The effectiveness of repellent compounds is not solely reliant on the biological function of the molecule but also on its method of dispersal. Conventional repellent delivery systems include aerosols, liquid emulsions and solid granules or powders, all of which have the advantage of immediate release of the AIs. However, these formulations do not provide great control over the rate of release or the stability of the actives and often result in the need for frequent reapplication and have off-target seepage into surrounding environments. Innovative formulations and compound delivery systems can greatly improve the efficacy and duration of current and future repellent chemistries while limiting off-target ecological effects.

**Figure 4:**
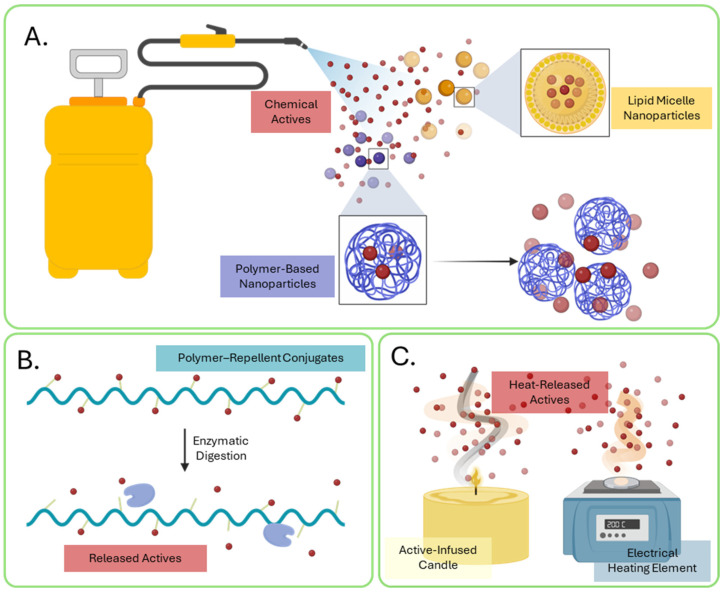
Novel controlled-release formulation and technology. (**A**) Polymer-based (blue) and lipid micelle (yellow) micro-encapsulation of chemical AIs (red) in nanoparticle formulations. AIs are released on degradation of the nanoparticles via heat, UV or physical disruption. (**B**) Polymer-repellent conjugates allow for temporally regulated release of covalently bonded AIs from polymer scaffolds using enzyme digestion. (**C**) Heat-based formulations increase volatility and effective distance of repellent AIs.

Research in nanoparticle and micro-encapsulations technologies aims to develop long-lasting repellent formulations using lipids and polymers to generate protective coatings around the active compounds and control their rate of release (Figure 4A, Table 1) [97,98]. Additionally, the nano- and micro-encapsulation of insect repellents would allow for novel modes of delivery based on the mechanisms and rate of degradation of the formulations. Photo-degradable polymers, for example, would allow for the solar-activated slow release of micro-encapsulated chemical repellents without the need for human interventions, with practical implications for protection against both disease vectors and agricultural pests [99]. Meanwhile, durable nanoparticle formulations could be integrated into gel-like matrices, paints and protective coatings for long-lasting efficacy [100,101,102]. Thus far, both lipid- and polymer-based formulations have demonstrated promising results for the sustained efficacy of repellent agents against both agricultural pests and disease vector insects [100,103,104]. Similarly, polymeric conjugates have also been explored as innovative structures to control the rate of repellent release and limit permeation into human skin (Figure 4B, Table 1). Polymer–repellent conjugates are synthesized by forming covalent bonds between polymeric carriers and repellent compounds, resulting in stable macromolecular structures that can be activated through chemical and biochemical methods [105]. In one study, the repellent para-menthane-3,8-diol (PMD) was esterified with acryloyl chloride into monomeric acryloyl-PMD (APMD) and subsequently co-polymerized with acrylic acid (AA) to form co-polymeric chains of poly(AA–co–APMD) [106]. In this example, the release of the PMD was activated by the enzymatic hydrolysis of the polymeric backbone via porcine liver esterases, which elicited the rapid release of the active within the first 24 h and gradual release for up to 5 days. Interest in polymer–repellent conjugates strongly prioritizes the applicability of these AIs for protection against biting insects while emphasizing the minimized permeability of potentially harmful AIs into human skin. While nano-/micro-encapsulation technologies and polymer conjugates offer promising mechanisms for the highly regulated release of repellent AIs, substantial work is still required to address regulatory, scalability and field performance challenges for large-scale implementation.

The thermal volatilization of chemical repellents has also been explored using various heat paradigms and has been adopted for commercial use in products such as Thermacell^®^ to increase the volatility of d-allethrin and metofluthrin using butane or electrical heating elements [107,108]. The effective range of low volatility compounds can be greatly enhanced by implementing heat, creating zones of repellency surrounding the vapor source and potentially allowing contact repellents to behave as spatial repellents (Figure 4C, Table 1). Historic strategies for repelling insects have included the use of plant burning in ancient societies as well as the use of candles containing botanical extracts such as citronella oil since the early 20th century [7,109]. While the volatilization of repellent compounds using heat is far from a novel concept, modern technological innovations have expanded the repertoire of available heat sources and chemical actives to create powerful repellent formulations. Moreover, recent studies have shown that the physiological activity of both longstanding and modern compounds, such as DEET and VUAAs, respectively, can be dramatically increased by induction coil heating [96,110,111]. Additionally, high temperatures can induce thermal degradation and thermolytic changes that alter the efficacy and utility of the original repellent molecule. While the thermolytic products may possess stronger repellent effects or greater spatial ranges than the original molecule, as seen with the breakdown of VUAAs into VUAIs [95,110], it should be noted that the thermal degradation of insect control agents can emit toxic and dangerous chemicals with implications for human and environmental health [112].

## 5. Concluding Remarks

Chemical strategies have long been used to combat the threats of insect populations to human health, food production and the environment. However, as global climates change, disease pathogens evolve and detrimental insects develop new ways to resist our current repertoire of chemical agents and the need for new and effective insect control strategies intensifies. Innovative, non-chemical insect control strategies—such as the exploitation of various male sterility approaches through Wolbachia infection and irradiation [113,114,115], genetic modifications to reduce vectorial capacity in insects [116] and increase pest resistance in crop plants [117], the lethal knockdown of essential genes with exogenous RNAi-based pest control [118], and the implementation of various mass-trapping techniques using attractant baits [119,120]—have been further explored in recent years and foreshadow long-lasting alternatives to traditional methods. While these strategies certainly offer legitimate pathways for disease vector control, rapid implementation on a global scale seems unlikely because of economic, environmental and legislative hurdles.

The development of novel chemical agents targeting under-exploited elements of the chemosensory systems that govern insect behavior represents an important component within an integrated pest/vector management toolbox against the growing threat of infectious disease transmission and food supply contamination by insects. Future directions for vectored disease prevention and securing food availability should prioritize the following key elements: (1) target-oriented repellent development, including the SAR and decoupling of olfactory receptors, (2) environmental impact assessment of emerging chemical strategies, (3) risk assessment of resistance development, (4) the accessibility of novel strategies to communities threatened by insect-related hardships. The design, development and deployment of formulations and dispersal technologies that maximize their safety, efficacy and duration remains a significant challenge to these next-generation technologies.

## Figures and Tables

**Table 1:** Comparison of contemporary repellent formulation technologies.

Delivery Technology	Functional Features	Field Applications	Advantages	Limitations
Nano-/micro- encapsulation	Lipid or polymer capsule containing actives.	Agricultural/industrial sprays.	Increased durability of actives.	Requires specialized equipment.
		Coatings/finishes for shipping containers.	Controlled release (temporal, UV, heat).	High cost of materials.
			Demonstrable application potential.	
Polymer– Repellent Conjugates	Actives covalently attached to polymer macromolecules.	Personal sprays and lotions against anthropophilic insects.	Increased durability/stability. Controlled release (enzymatic).	Requires enzymatic digestion to release actives (possible skin irritant).
			Reduced skin permeation.	Early exploratory phase.
Thermal Volatilization	Heat source used to increase the volatility of actives.	Spatial protection against agricultural/industrial pests and disease vectors.	Increased volatility of high-molecular-weight actives.	Thermolytic breakdown products of actives may be inactive or toxic.
			Does not require specialized equipment, candles and electric heating elements are sufficient.	
			Large zones of protection.	

## Data Availability

No new data were created or analyzed in this study. Data sharing is not applicable to this article.

## Abbreviations

The following abbreviations are used in this manuscript:

DDT	Dichloro-diphenyl-trichloroethane
DMP	Dimethyl phthalate
DBP	Dibutyl phthalate
DEPA	N,N-diethyl phenylacetamide
DEET	N,N-diethyl-m-toluamide
MOA	Mechanism of action
ORN	Olfactory receptor neuron
OR	Olfactory receptor
GR	Gustatory receptor
GRN	Gustatory receptor neuron
VUAA	Vanderbilt University Allosteric Agonist
VUAI	VUAA-based active intermediate
MCH	3-methyl-2-cyclohexen-1-one
EBF	(E)-β-Farnesene
PMD	*Para*-menthane-3,8-diol
AA	Acrylic acid
AMPD	Acryloyl-PMD
AI	Active ingredient
USDA	United States Department of Agriculture
